# A case report of fulminant endophthalmitis caused by *Streptococcus dysgalactiae* in a patient with traumatic corneal laceration

**DOI:** 10.1186/s12886-020-01511-z

**Published:** 2020-06-18

**Authors:** Yong Woo Lee, Kyung Min Koh, Kyu Yeon Hwang, Young A. Kwon, Dong Won Lee, Sang Wroul Song, Byoung Yeop Kim, Kook Young Kim

**Affiliations:** Department of Cornea and Refractive Surgery, Kim’s Eye Hospital, 136, Yeongshin-ro, Yeongdeungpo-gu, Seoul, South Korea

**Keywords:** Corneal laceration, Endophthalmitis, Enucleation, *Streptococcus dysgalactiae*

## Abstract

**Background:**

To report a case of enucleation caused by *Streptococcus dysgalactiae* endophthalmitis after traumatic corneal laceration.

**Case presentation:**

A 69-year-old man with history of retinal detachment treated with vitrectomy and subsequent cataract surgery presented with traumatic corneal laceration while cutting grass. Appropriate repair of corneal laceration and intravitreal antibiotics (vancomycin, ceftazidime) injection was performed. *S. dysgalactiae* which was sensitive to the conventional antibiotics (Ampicillin, Ceftriaxone, Levofloxacin, etc.) detected by aqueous culture. One day following primary closure, the patient developed a complete hypopyon and vitreous membranes. Despite vigorous systemic and intravitreal antibiotics administration with vitrectomy, endophthalmitis was not controlled and patient’s ocular pain was increased. The vitreous culture was also positive for *S. dysgalactiae*. Finally, total enucleation was performed 9 days after trauma due to fulminant endophthalmitis with severe scleritis.

**Conclusion:**

Progression of traumatic endophthalmitis associated with *S. dysgalactiae* can be fulminant. Sufficient warning to patient about enucleation and intensive care is needed in the case of this infection.

## Background

Endophthalmitis is a rare, but it can lead to irreversible complication affected to visual acuity if not treated promptly. Endophthalmitis can be categorized as endogenous or exogenous according to the infectious route. The incidence of post-traumatic exogenous endophthalmitis after open-globe injury has been reported to be 0 to 16.5%, and risk factors of visual loss include a retained intraocular foreign body, ruptured crystal lens, and delayed treatment [1]. Fulminant endophthalmtis is rare, but it can lead to evisceration or enucleation due to rapid progression. Organisms associated with fulminant endophthalmitis are known to involve *Pseudomonas*, *Clostridium* and *Streptococcus* species [2]. Group G ß-hemolytic *Streptococcus dysgalactiae* is occasionally causative organisms of endogenous endophthalmitis especially associated with endocarditis [3]. Here we report a case of post-traumatic endophthalmitis due to *S. dysgalactiae* that progressed to fulminant endophthalmitis, which was not expected. This is the first case of exogenous endophthalmitis with *S. dysgalactiae* after penetrating globe trauma to our knowledge.

## Case presentation

**Fig. 1:**
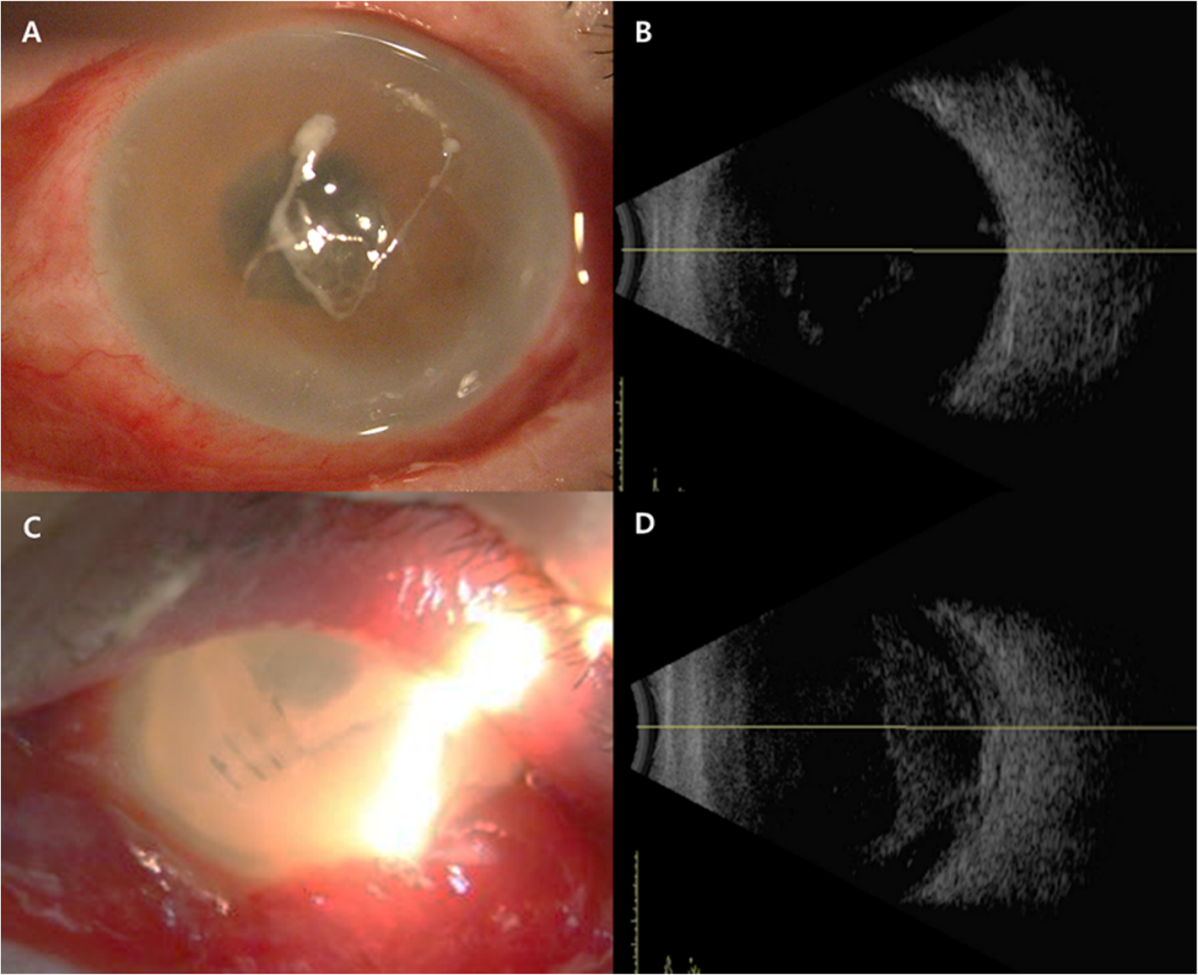
Anterior segment photographs and B-scan sonographs. **a:** About 5 mm central corneal full thickness laceration was presented with little anterior chamber reaction at initial presentation. **b:** Vitreous opacity was found in B-scan at initial presentation. **c:** Severe conjunctival chemosis and anterior chamber reaction were found on postoperative 4 days, but corneal suture was intact. **d:** Vitreous infiltration was aggravated and scleral thickness was increased in B-scan finding

**Fig. 2:**
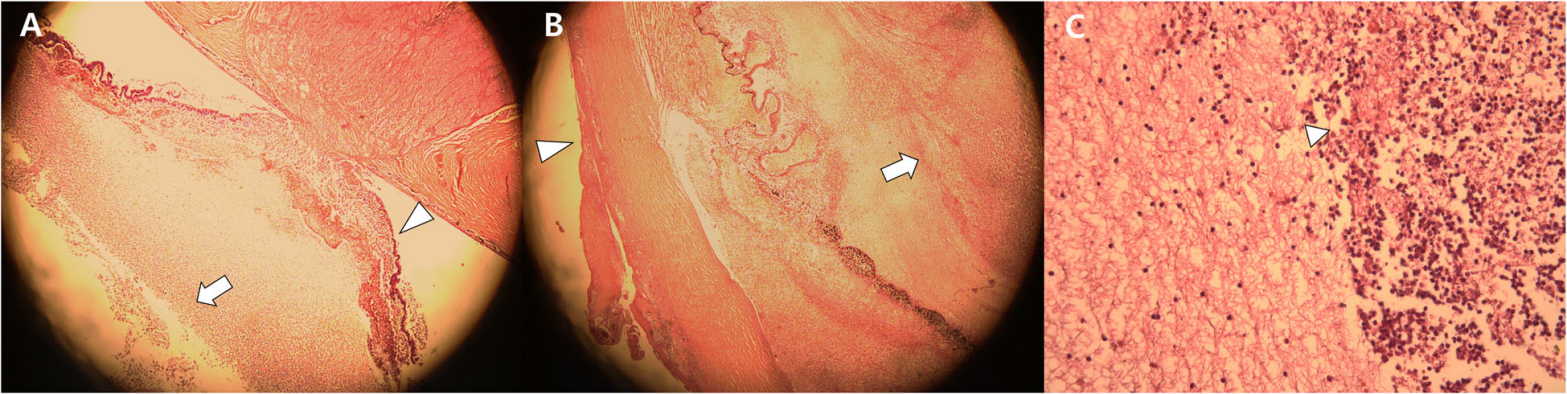
Histologic images after enucleation. **a:** Retinal detachment (white arrow head) was found and many inflammatory cells (white arrow) were in vitreous cavity with hematoxylin and eosin stain. (× 40 magnification) **b:** Inflammatory cells were in sclera (white arrow head) and in vitreous cavity (white arrow). (× 40 magnification) **c:** many neutrophils(white arrow head) were found in vitreous cavity. (× 400 magnification)

A 69-year-old male patient with history of vitrectomy for retinal detachment and cataract surgery presented with traumatic corneal laceration of the left eye secondary to a high velocity grass cutter injury that is suspected of being hit by small stones splattered during grass cutter operation. There was no foreign body through computed tomography scan and slit-lamp examination, but siedel test was positive with 5 mm full thickness corneal laceration (Fig. 1a). There was slightly anterior chamber reaction. Best corrected visual acuity was finger counting and intraocular pressure could not be measured in the left eye. Corneal primary closure and intravitreal antibiotic injection (vancomycin 1.0 mg/0.1 mL, ceftazidime 2.5 mg/0.1 mL) were performed and some anterior chamber fluid was collected and cultured. The globe was adequately closed on the first postoperative day, and there were no vitreous membranes on B-scan ultrasonography. However, increased inflammation was detected in the anterior chamber and there were vitreous membranes consistent with a diagnosis of endophthalmitis. Gram positive cocci were detected in the specimens taken from the anterior chamber, and antibiotic susceptibility test and bacterial identification were performed. On postoperative day 2, anterior chamber and intravitreal inflammation were increased in the B-scan and anterior segment findings, and *S. dysgalactiae* (Ampicillin<=0.25S, Cefotaxime<=0.12S, Ceftriaxone<=0.12S, Clindamycin<=0.25S, Erythromycin<=0.12S, Levofloxacin = 2S, Linezolid<=2S, PenicillinG<=0.06S, Trimethoprim/Sulfamethoxazole<=10S, Vancomycin = 0.25S, Tetracycline> = 16R) was identified in aqueous sample. On postoperative day 4, total vitrectomy was planned but was impossible due to anterior chamber intussusception and excessive abscess in the vitreous body. Core vitrectomy, intravitreal antibiotics injection, and vitreous specimen was taken. *S. dysgalactiae* was cultured in both anterior and vitreous specimens. The patient received topical (Moxifloxacin 5 mg/mL, fortified tobramycin 14 mg/mL and fortified ceftazidime 50 mg/mL), intravitreal (vancomycin 1.0 mg/0.1 mL, ceftazidime 2.5 mg/0.1 mL) and systemic (intravenous clindamycin 300 mg/2 mL/12 h and oral cefixime 200 mg/12 h) antibiotics. While sensitivity testing suggested that the isolated organism was susceptible to these agents, the infection remained poorly controlled. Then, enucleation was performed because the infection was poorly controlled, and the patient had increasing pain with no useful vision and a poor visual prognosis on postoperative 9 days. Enucleation specimen revealed total vitreous abscess and severe scleritis (Fig. 2).

## Discussion and conclusion

This case report describes a case of *S. dysgalactiae* endophthalmitis in the setting of prior penetrating trauma. Despite aggressive treatment including topical, intravitreal, and systemic antibiotics as well as vitrectomy, the outcome was unfavorable, and the globe could not be salvaged. It is well known that early surgical wound closure and restoration of globe integrity as well as broad-spectrum prophylactic antibiotic treatment may improve visual outcomes following penetrating globe injuries. Broad-spectrum antibiotics treatments should be started before results of bacterial identification culture are available. Intravitreal antibiotic treatment with ceftazidime and vancomycin are generally chosen for the first treatment option like this case [1]. In the antibiotic susceptibility test, all used antibiotics (moxifloxacin, fortified tobramycin, fortified ceftazidime, vancomycin, ceftazidime, clindamycin, cefixime) were within the sensitivity range in this case. A vitrectomized eye of a patient due to retinal detachment in the past, tend to induce the influx of microorganisms or foreign substances faster than gel-form vitreous when the globe ruptures. In addition, liquefied vitreous or vitrectomized eye are known to have a faster clearance of intravitreal antibiotics [4, 5], so it is possible that the injected antibiotics could not easily reach the concentration to suppress bacterial virulence. For these reasons, we administered the intravitreal antibiotics immediately after the primary repair of cornea. However, endophthalmitis caused by *S. dysgalactiae* in this case was did not respond to vancomycin and ceftazidime and progressed too fast within 1 week.

*S. dysgalactiae* is a common colonized bacteria of the pharynx, gastrointestinal and female genital tracts, and the skin. Its transmission usually occurs through direct contact with aerosolized respiratory secretions or, skin contact with infected skin lesions [6]. Recently, the number of infection cases with *S. dysgalactiae* is increasing [7].

There are several reports of endogenous endophthalmitis with *S. dysgalactiae*. Most common associated disease is infective endocarditis, accounting for about half of all cases, and other causes are various such as skin infection, dental procedure and osteomyelitis [3, 7–12]. Suemori et al. reported one case of endogenous endophthalmitis with *S. dysgalactiae*, which following emergency cardiac surgery, improved with intravitreal and subconjunctival vancomycin and meropenem, topical levofloxacin, and intravenous gentamicin and penicillin G; however, the best-corrected visual acuity was not improved [13]. Similarly, Hagiya et al. presented the patient with systemic infection of *S. dysgalactiae* involving eye, mediastinum, hear, brain and multiple joint after valve replacement surgery [3]. They reported that they could not save the globe and this organism should be causative invasive pathogen especially in the elderly aged patients. Gupta et al. showed delayed endophthalmitis after endocarditis and *S. dysgalactiae* could be associated with poor visual prognosis [10].

However, epidemiology of exogenous endophthalmitis with *S. dysgalactiae* could be probably underestimated in ophthalmologic infectious aspect. Kaliamurthy et al. [14] reported three patients with a history of uncomplicated cataract surgery developed exogenous endophthalmitis due to *S. dysgalactiae*. Intravitreal (cefazolin, amikacin) and topical (ofloxacin, tobramycin) antibiotic treatments resulted in resolution of infection in all three patients. They reported that all three patients had no underlying disease and no history of previous ocular surgery. In our case, the patient had history of previous ocular surgery and cause of infection was penetrating corneal laceration. We presumed that these factors could affect to poor prognosis of endophthalmitis compare with uncomplicated cataract surgery cases. It is significant that *S. dysgalactiae* was positive at aqueous and vitreous culture, then it is apparent that this organism was cause of fulminant endophthalmitis in our case. In addition, progression of disease was very severe and rapid, so that we had to removal of globe instead of evisceration due to severe scleritis.

Traumatic exogenous endophthalmitis infected with *S. dysgalactiae* may show a fulminant course regardless of antibiotic susceptibility. Sufficient warning to patient about poor prognosis of this infection and possibility of enucleation is essential. Prompt surgical treatment can save the globe and prevent the spread of infection to adjacent tissues of patients infected with *S. dysgalactiae*.

## Data Availability

All the data supporting the conclusions of this article is included in the present article.
